# Comparative studies on structure of the floral nectaries and the abundance of nectar production of *Prunus laurocerasus* L.

**DOI:** 10.1007/s00709-019-01412-z

**Published:** 2019-07-17

**Authors:** Mirosława Chwil, Mikołaj Kostryco, Renata Matraszek-Gawron

**Affiliations:** Department of Botany and Plant Physiology, University of Life Sciences in Lublin, Akademicka 15, 20-950 Lublin, Poland

**Keywords:** Anatomy, Cherry laurel, Floral biology, Micromorphology, Sugar components, Ultrastructure

## Abstract

There is very scanty information concerning the floral nectary structure and nectar secretion in *Prunus laurocerasus* L. Therefore, the aim of the study was to determine the micromorphology, anatomy and ultrastructure of nectaries; the abundance of nectar production; and the quantitative and qualitative composition of sugars contained in the nectar of two *P. laurocerasus* cultivars: ‘Schipkaensis’ and ‘Zabeliana’. The nectary structure was studied using light, fluorescence, scanning and transmission electron microscopy techniques. The nectar sugars were analysed with HPLC. The ‘Schipkaensis’ had longer inflorescences with a larger number of flowers and a longer perianth than ‘Zabeliana’. The micromorphological structure of the nectaries in ‘Schipkaensis’ exhibited denser (approx. 39%) and larger (approx. 50%) stomata and thicker (approx. 13%) cuticular striae forming wider bands (approx. 26%) than in ‘Zabeliana’. The results provide new data on the micromorphology, anatomy and ultrastructure of these floral nectaries. Nectary cuticle ornamentation as well as the size, type and density of stomata and stomatal complex topography can have a diagnostic value in *Prunus*. The nectar sugar weight indicates a significant apicultural value of the cherry laurel, especially in the case of ‘Schipkaensis’. Cherry laurel is an entomophilous species recommended for cultivation in nectariferous zones and insect pollinator refuges; however, climatic conditions eliminating the invasiveness of these plants should be considered.

## Introduction

*Prunus laurocerasus* L. (Prunoideae-Rosaceae) is an evergreen shrub reaching a height of 10 m (Lee and Wen 2001; Öztürk and Ölçücü 2016). It is native to the Black Sea region, south-west Asia and southeast Europe (Kolayli et al. 2003; Sulusoglu et al. 2015). In the natural environment, the species colonises Serbia, Bulgaria, the Caucasus and Iran. It grows on rocky slopes and in scrubs and forest undergrowth (Kolayli et al. 2003; Sulusoglu 2011). In Central Europe, the cherry laurel has been cultivated since the fifteenth century. In Germany, it was introduced for cultivation in 1663 (Sukopp and Wurzel 2003).

Two *P. laurocerasus* cultivars, i.e. Schipkaensis’ and ‘Zabeliana’ are well acclimatised in Poland (Seneta and Dolatowski 2012). With their decorative leaves and abundantly flowering inflorescences, they are recommended for planting in parks and gardens (Marco et al. 2008; Cameron et al. 2014). In some parts of Europe, e.g. in England, Germany, Switzerland and Italy, the shrubs can pose a threat as invasive plants (Hättenschwiler and Körner 2003; Sukopp and Wurzel 2003; Kautz et al. 2017).

### Apicultural value

The abundantly blooming *P. laurocerasus* shrubs (April–May) provide pollinating insects with nectar and pollen reward for 3–4 weeks (Percival 1955; Williams et al. 1993; Yilmaz 2016). Their flowers were mainly visited by *Apis mellifera*, which collected nectar throughout the day, with maximum activity at noon (Percival 1955; Gupta et al. 1990). There are various types of honey from the nectar *Prunus* type. Furthermore, a large part of *Prunus* pollen grains in bee loads has been found (Wróblewska and Stawiarz 2015).

### Nectaries in representatives of the genus *Prunus*

Nectar in different *Prunus* species is secreted by extra-floral and floral nectaries. Extra-floral and floral nectaries are found in many species of the genus: *P. avium* (Pulice and Packer 2008), *P. laurocerasus* (Winnall 2012), *P. persica* (Mathews et al. 2009), *P. sellowii* (Machado et al. 2008) and *P. serotina* (Tilman 1978). In different taxa, these glands are located on the stipules, petiole, lamella and petiole base or on several organs in some species (Pemberton 1990; Pemberton and Lee 1996; Chin et al. 2013). The cherry laurel has extra-floral nectaries at the petiole and lamella base and on the lamella margin and surface (Kalkman 1965).

The floral nectaries in the subfamily Prunoideae are located on the adaxial surface of the receptacle between the filament base and the basal part of the ovary (Bernardello 2007; Radice and Galati 2003; Chwil 2013). They have been classified as receptacular nectaries (Bernardello 2007; Fahn 1979). This type of nectary has been described in several representatives of *Prunus*: *P. avium*, *P. armeniaca*, *P. cerasus*, *P. domestica*, *P. dulcis* and *P. persica* (Orosz-Kovács and Apostol 1996; Radice and Galati 2003; Farkas and Zajácz 2007; Chwil 2013).

#### Micromorphology

The floral nectaries in various taxa of the genus differ in the size, topography, type of stomata and cuticle ornamentation (Fahn 1979; Chwil and Weryszko-Chmielewska 2011; Chwil 2013). Striated cuticular ornamentation on the nectary epidermis surface was observed in *P. armeniaca*, *P. persica*, *P. cerasus* and *P. amygdalus*, whereas *P. domestica* was characterised by reticulate ornamentation (Orosz-Kovács 1993; Orosz-Kovács and Apostol 1996; Radice and Galati 2003; Chwil 2013). In terms of cuticle ornamentation, nectaries are divided into mesomorphic glands with sparse striae and xeromorphic ones with denser striae and a thicker cuticle layer (Orosz-Kovács and Apostol 1996; Orosz-Kovács et al. 1996, 1998). The stomata in the epidermis of the *P. persica* nectary were located below guard cells. The stomata and microchannels in the cuticle were involved in nectar secretion onto the surface of the nectary. During this process, the stomata in the peach were open. Additionally, there were one- or two-celled non-glandular trichomes in the epidermis of the *P. persica* nectary (Radice and Galati 2003; Chwil 2013).

Anatomy. The literature provides insufficient information about the anatomical structure of floral nectaries in *Prunus*. The nectary gland in several species from this genus was composed of a single layer of epidermis cells, several layers of nectary parenchyma and sub-nectary parenchyma. The parenchyma cells were smaller than the cells of the nectary epidermis and sub-nectary parenchyma. The nectary cells of several *Prunus* species contained numerous plastids with starch granules (Orosz-Kovács 1993; Radice and Galati 2003; Chwil 2013).

#### Ultrastructure

The ultrastructure of floral nectaries in representatives of *Prunus* has been poorly investigated to date. There is scanty information in this field in *P. armeniaca*, *P. avium*, *P. cerasus*, *P. domestica* and *P. persica* (Radice and Galati 2003; Liu and Zhao 2011; Chwil 2013). Since there is no information about the floral nectary structure and nectar secretion in *P. laurocerasus*, we have undertaken an attempt to complete the data.

The aim of the study was to determine the micromorphology, anatomy and ultrastructure of the floral nectaries, nectar secretion and the quantitative and qualitative composition of sugars in the nectar of two *P. laurocerasus* cultivars: ‘Schipkaensis’ and ‘Zabeliana’.

Studies on the nectary structure combined with investigations of secretion and pollination processes are helpful in explaining onto- and phylogenesis and are important for plant taxonomy. The cuticular ornamentation and the topography of stomata on the surface of the *P. laurocerasus* nectary epidermis are helpful in differentiation between closely related taxa of this genus.

## Materials and methods

### Plant material

The research was carried out in 2016–2018 on two *Prunus laurocerasus* cultivars: ‘Schipkaensis’ and ‘Zabeliana’. The observations of cherry laurel flowering were conducted in the Botanical Garden of Maria Curie Skłodowska University in Lublin located in the east-south part of Poland (51° 16′ N, 22° 30′ E). Flowers for the analysis of the nectar content and examination of the nectary ultrastructure were collected in the full bloom stage.

### Fixation of the material

Fragments of nectaries were sampled from the flowers located in the middle portion of inflorescences in the initial phase of nectar secretion. Sections were fixed in 4% glutaraldehyde for 6 h at room temperature and in 0.01 M phosphate buffer, pH 7.0, for another 48 h at 4 °C. The nectary samples were subjected to respective treatments for:

- Observations under a scanning electron microscope—fixed samples were dehydrated in an acetone series: 15, 30, 50, 70, 90 and 99.5% and twice in anhydrous acetone for 15 min at room temperature.
- Preparation of semi-thin and ultrathin sections—the plant material was contrasted in a 1.5% osmium tetraoxide solution for 1.5 h. After rinsing with distilled water, a 0.5% aqueous uranyl acetate solution was applied for 2 h at room temperature. After double rinsing with distilled water, the nectary fragments were dehydrated for 15 min in an ethyl alcohol series of subsequent concentrations: 15, 30, 50, 70, 90, 96 and 99.8% and subjected to absolute ethanol twice. The dehydrated samples were embedded in Spurr low viscosity resin and polymerised at 60 °C for 48 h.

### Microscopy

The structure of the floral nectaries was analysed with the use of bright-field microscopy as well as fluorescence, scanning and transmission electron microscopy techniques.

#### Light microscopy

The micromorphology of the nectaries as well as floral elements in the beginning and full phase of nectar secretion was compared using a SMT 800 (SM) stereoscopic microscope equipped with a Nikon Colpix 4300 camera. Longitudinal semi-thin sections were prepared for the analyses of the anatomical features of the nectaries. The 0.8–1.0-μm-thick sections were cut with a glass knife using a Reichert Ultracut S microtome. Nectary fragments were stained with 1% toluidine blue and 1% azure II (1:1) at a temperature of 60 °C for 5 min. The sections were rinsed with distilled water and 5% ethyl alcohol and dried. The location of starch granules in the plastids and the presence of other polysaccharides in the cell walls were identified with the PAS reaction (Nevalainen et al. 1972). Observations of the nectary structure, measurements of selected anatomical features and photographic documentation were made using a Nikon Eclipse 90i clear field microscope equipped with a Nikon 5Mpix CCD camera.

#### Fluorescence microscopy

To examine the cuticle layer, the semi-thin sections of the fixed material were placed in a drop of fluorochrome (0.01% auramine O) (Heslop-Harrison 1977). The preparations were embedded in a 50% glycerol solution. Comparative studies were performed with the use of a Nikon Eclipse 90i fluorescence microscope equipped with FITC filters: EXP. 465-495, DM 505 and BA 515-555.

#### Scanning electron microscopy

Fragments of nectaries dehydrated in the acetone series were critical point dried in liquid CO_2_ in an Emitech K850 dryer and sputter-coated with gold using an Emitech K550X sputter coater. Micromorphological observations of the nectary epidermis surface and photographic documentation were made using a Tescan Vega II LMU scanning electron microscope (SEM).

#### Transmission electron microscopy

Ultrathin 70-nm sections were stained for 40 min with an 8% uranyl acetate solution in 0.5% acetic acid. After double rinsing with distilled water (10 min), Reynolds reagent was applied for 15 min (Reynolds 1963). After rinsing with water, the sections were dried. The ultrastructure of the nectary cells was viewed using a FEI Tecnai Spirit G2 transmission electron microscope (TEM).

### Microscopic morphometric measurements

The following morphometric measurements of selected floral traits were carried out as part of the analysis of the two *P. laurocerasus* cultivars: (i) diameter of the nectary, (ii) micromorphology of the nectary epidermis surface (length and width of stomata, surface area of stomata, length and width of the aperture between cuticular ledges, number of stomata per 1 mm^2^ of the epidermis, minimum and maximum diameter of the stomatal complex, surface area of the stomatal complex, thickness of cuticular striae for stomatal complex and the other epidermal cells, distance between cuticular striae for stomatal complex and the other epidermal cells, number of striae on the cell surface for stomatal complex and the other epidermal cells, width of the band of cuticular striae for stomatal complex and the other epidermal cells, diameter of the other epidermal cells), (iii) anatomy of the nectary (height and width of nectary epidermis cells, thickness of the parenchyma cell layer in the nectary, number of nectary parenchyma layers, diameter of nectary parenchyma layers, thickness of the sub-nectary parenchyma cell layer in the nectary, number of sub-nectary parenchyma layers, diameter of sub-nectary parenchyma layers, height and width of abaxial epidermis cells, thickness of the nectary gland, thickness of the receptacle, thickness of the receptacle with the nectary) and (iv) ultrastructure of nectary epidermis cells (thickness of the cuticle layer, thickness of the remaining part of the outer periclinal cell wall without the cuticle, thickness of the outer periclinal cell wall, thickness of the inner periclinal cell wall, thickness of the anticlinal cell wall).

The measurement of each analysed feature was performed in 16 replications. The comparative morphometric micromorphological, anatomical and ultrastructural studies were carried out using Nikon NIS-Elements computer-aided analysis software version 3.0 Advance Research.

### Abundance of nectar secretion

Bursting flower buds that were intended for nectar collection at the flowering stage were labelled and protected against insect visits using airy tulle covers. Nectar was collected with the pipetting method from 9:00 to 10:00 h. The amount of nectar secreted by 5–10 flowers throughout their lifespan (*n* = 12) constituted a sample. The nectar weight from a known number of flowers was determined using an analytical balance and converted into a single flower. The percent content of sugars present in the nectar was determined with the use of an Abbe refractometer (RL-1 PZO). The nectar weight in the flowers and the percent proportion of sugar in the nectar were used for calculation of the weight of sugars in the nectar of the analysed cultivars.

### Quantitative and qualitative composition of sugars in the nectar

The quantitative and qualitative analyses of sugars contained in the nectar collected throughout the lifespan of the analysed flowers of both studied cultivars were performed with the high-performance liquid chromatography (HPLC) method proposed by Bogdanov et al. (1997) and modified by Rybak-Chmielewska and Szczęsna (2003) and Rybak-Chmielewska (2007). Sugars were determined with a Shimadzu liquid chromatograph. Individual sugars in the nectar were identified qualitatively by comparison of their retention times in the standard solution. The quantification was performed by comparison of the area of peaks of the individual sugars in the standard solution and in the nectar solution. The percent content of glucose (G), fructose (F) and sucrose (S) and the ratio of the sugars (S/F + G) were determined. The quantitative and qualitative analysis of sugars contained in the nectar for each cultivar was performed in three replications.

### Statistical analysis

The significance of the differences in selected features of floral nectary, epidermal micromorphology, tissue anatomy and ultrastructure of floral nectary cells as well as abundance of nectar production was analysed with the Statistica 6.0 integrated statistical software package. One-way analysis of variance (ANOVA) with post-hoc Tukey HSD (honestly significant difference) test was performed. Statistical inference was carried out at the level of significance of *P* = 0.05. Furthermore, for the data concerning abundance of nectar secretion as well as quantitative and qualitative composition of sugars in the nectar, which are presented in the figures, standard deviations (SD) of the mean values are given. Standard deviations of the mean values are also given for the data obtained from microscopic-morphometric measurements presented in the tables.

## Results

**Fig. 1:**
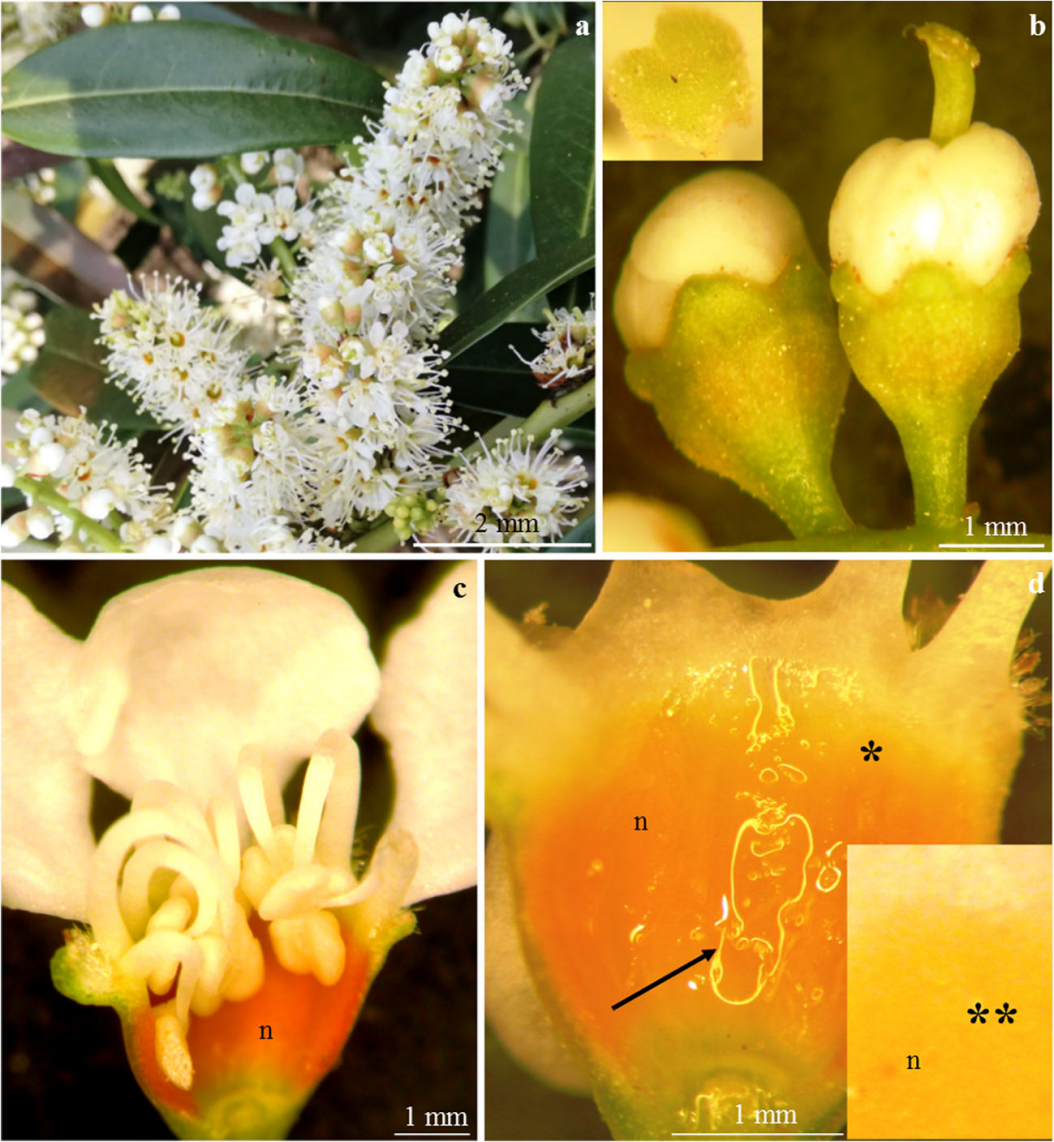
**a–d***Prunus laurocerasus* ‘Schipkaensis’ inflorescence, flowers, and nectary. **a** Raceme with buds in different developmental phases. **b** Bursting buds with visible protogyny. **c** Bursting bud (onset of nectar secretion), visible intense orange nectary (n). **d** Bright orange-yellow (asterisk) and yellow colour (two asterisks) of the nectary (n) at a later stage of nectar secretion, visible nectar (arrow). **b** SM. **c**, **d** LM

**Fig. 2:**
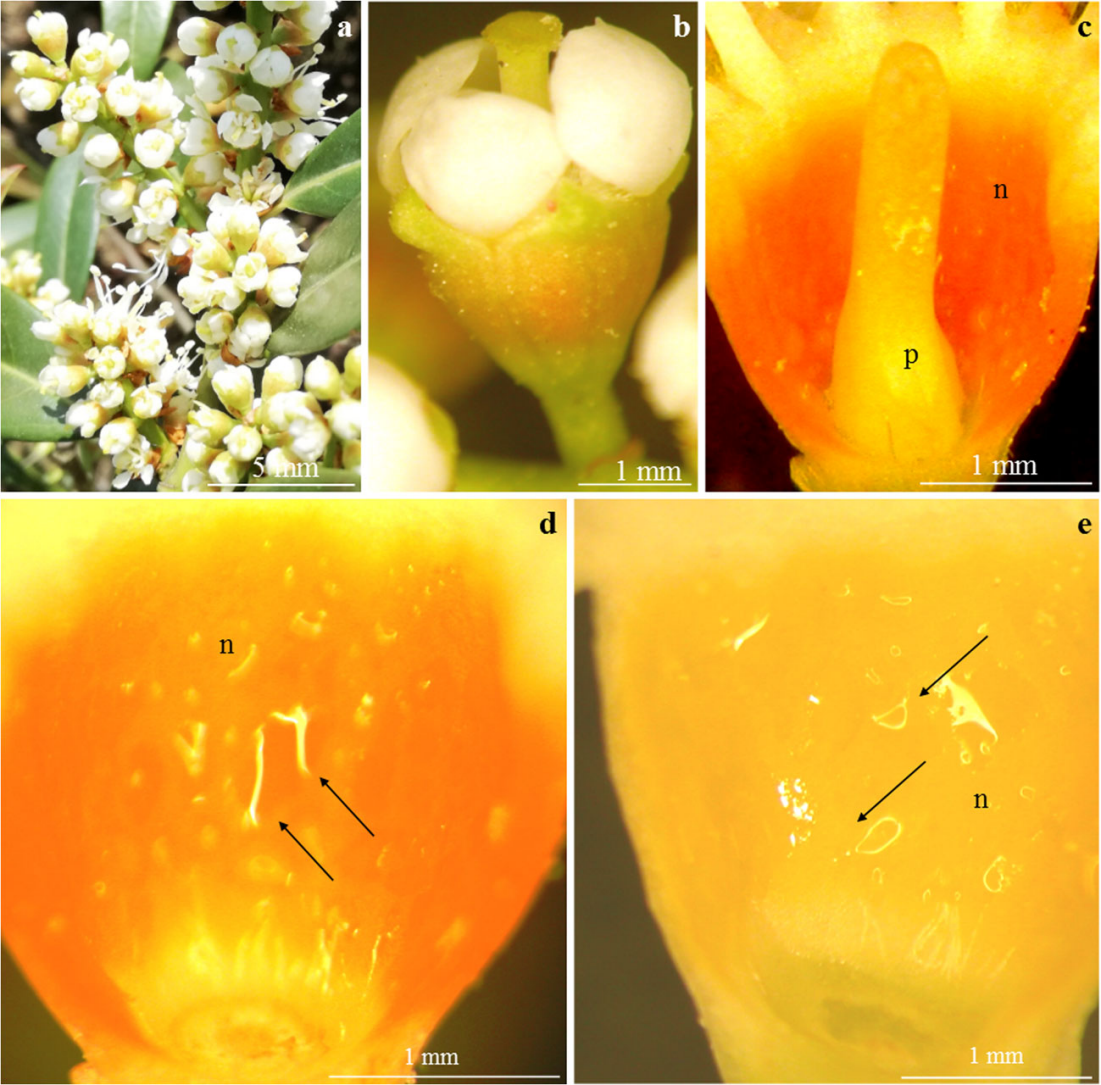
**a–e***Prunus laurocerasus* ‘Zabeliana’ inflorescence (**a**), flowers (**b**) and nectary (**c**–**e**). **a** Raceme with buds in different developmental phases. **b** Bursting bud with visible protogyny. **c** Intense orange colour of the nectary (n) at the beginning of nectar secretion (bursting bud), visible upper pistil (p). **d** Small and large drops of nectar (arrow) on the nectary surface (n) in the flowers at the onset of anthesis, visible intense orange colour of the active secretory cells. **e** Bright yellow colour of the nectary (n) in a blooming flower (full nectar secretion phase), visible accumulated nectar (arrow). **b** SM. **c**–**e** LM

The flowers of *P. laurocerasus* ‘Shipkaensis’ and ‘Zabeliana’ formed a raceme (Figs. 1a and 2a) and were characterised by protogyny (Figs. 1b and 2b). The elements of the perianth and the stamens were located on a concave, cup-shaped receptacle (Figs. 1c and 2c).

### Location and colour of the nectary

The nectaries in the flowers of the examined *P. laurocerasus* cultivars were located on the adaxial side of the receptacle. Active secretory cells were found on the receptacle between the basal portion of the ovary and the base of stamen filaments (Figs. 1c and 2c). The upper part of the nectary almost reached the filament base (Figs. 1d and 2c) .

The nectaries in the flowers of the analysed cultivars differed in their colour depending on the developmental stage. In the bud burst stage, the nectary was intense orange (Figs. 1c and 2c). The colour of the nectaries determined by the number of chromoplasts changed during the bud development phases and during flowering (Figs. 1d and 2d). The orange colour observed in the presecretory phase turned into light yellow in the final phase of nectar secretion (Figs. 1d and 2e). The nectary diameter ranged from 2.5 mm (‘Schipkaensis’) to 2.7 mm (‘Zabeliana’) (Table 1).

**Table 1:** Nectary diameter and characteristics of the stomata and stomata complex in nectary epidermis of two studied *P. laurocerasus* cultivars

Tested feature			Cultivar			
			‘Schipkaensis’		‘Zabeliana’	
			Min.–max.	Mean ± SD	Min.–max.	Mean ± SD
Nectary diameter		(mm)	1.98–2.82	2.51 ± 0.34a	2.33–3.12	2.74 ± 0.33a
Stomata						
Length	of stomata	(μm)	13.37–17.94	15.60 ± 1.57a	9.12–14.36	11.73 ± 1.49b
Width			7.61–15.12	12.18 ± 1.94a	5.60–8.40	7.11 ± 0.94b
Surface area		(μm^2^)	92.89–166.98	122.28 ± 24.97a	46.07–94.56	64.41 ± 17.85b
Number of stomata per 1 mm^2^		(pc.)	46.44–99.14	71.01 ± 13.85a	32.08–53	45.23 ± 9.34b
Length	of the aperture between cuticular ledges	(μm)	4.92–8.03	6.04 ± 0.96a	4.42–6.39	5.42 ± 90.76a
Width			3.21–5.72	4.13 ± 0.86a	2.26–3.99	3.03 ± 0.58a
Stomatal complex						
Diameter of the stomatal complex	Min.	(μm)	47.03–73.06	61.02 ± 10.27a	51.32–76.05	66.24 ± 8.17a
	Max.		54.16–93.75	71.07 ± 13.38a	54.80–87.64	70.37 ± 8.77a
Surface area of the stomatal complex		(μm^2^)	2196.49–3963.62	3179.53 ± 628.58a	2812.31–4368.56	3504.93 ± 449.52a

The measurement of each analysed feature was performed in 16 replications (*n* = 16). For each tested features, means with the same small letter do not differ significantly among two studied cultivars (LSD post-hoc Tukey HSD test after univariate ANOVA, *P* < 0.05)

*± SD* standard deviation of means

### Micromorphology of the nectary epidermis surface

#### Stomata

**Fig. 3:**
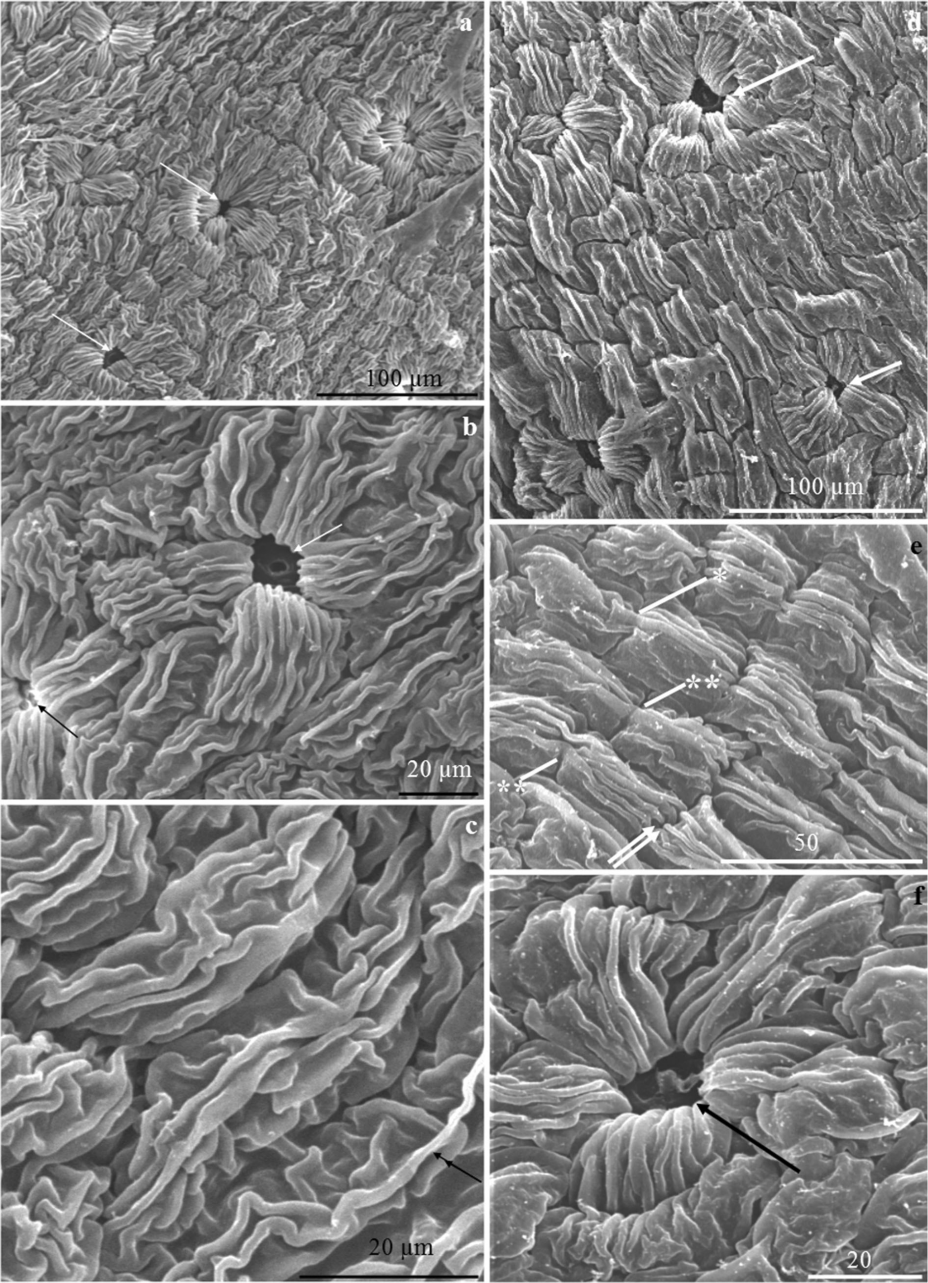
**a–f***Prunus laurocerasus* ‘Schipkaensis’ (**a**–**c**) and ‘Zabeliana’ (**d**–**f**) nectary epidermis. **a** Striated cuticular ornamentation, visible stomata (arrow) evenly distributed on the nectary surface. **b** Open stomata (arrow), located below other nectary epidermis cells, visible radial arrangement of cells in the stomatal complex with parallel or intertwined arrangement of cuticular striae. **c** Corrugated cuticular striae arranged along the longer cell axis of other epidermis cells sometimes with long major striae (double-headed arrow). **d** Striated cuticular ornamentation, visible stomata (arrow). **e** Cuticular striae in parallel arrangement sometimes with a slight bend maintaining continuity (asterisk) with the wall or forming a bend (two asterisks) over the anticlinal wall or convergent with the anticlinal wall (two arrows), visible striation along the longer axis of the cells. **f** Radial pattern of cuticular striae on the surface of guard cells. SEM

In *P. laurocerasus*, nectar was secreted onto the nectary surface through stomata (Fig. 3a, d). The stomata were distributed evenly in the nectary epidermis between the filament base and the basal ovary portion. The stomata were present below the other epidermis cells (Fig. 3b, f). The stomata had a narrow (3–4 μm) and short (5–6 μm) aperture with a cuticular ledge (Fig. 3b). The size of stomata in epidermal nectary cells in ‘Schipkaensis’ was significantly higher than in ‘Zabeliana’. Their length, width and surface were 16 μm, 12 μm and 122 μm^2^, respectively in the former cultivar, and 12 μm, 7 μm and 64 μm^2^, respectively, in the latter. There were 45 and 71 stomata per 1 mm^2^ of the ‘Zabeliana’ and ‘Schipkaensis’ epidermis, respectively. The differences in the number of stomata between the cultivars were significant. The stomata exhibited different degrees of opening. They were open, semi-open (Fig. 3b) and closed (Fig. 3f). The stomata were surrounded by 4–6 radially arranged cells, suggesting the actinocytic type (Fig. 3b, f). Together, they formed a stomatal complex with a diameter of 61–71 μm in the epidermis of ‘Schipkaensis’ and 66–70 μm in ‘Zabeliana’. The surface area of this complex in the analysed cultivars was in the range of 3505–3180 μm^2^ (Table 1).

#### Cuticle ornamentation

The entire surface of the nectary epidermis from the ovary base to the basal portion of filaments exhibited cuticular striation with varied arrangement of striae.

#### Stomatal complex

The stomata were located below the level of the other epidermis cells. The number of cuticular striae on the surface of the stomatal complex ranged from 4 to 10. The thickness of the striae in the examined cultivars was in the range of 1.7–1.9 μm. The distance between the striae on the surface of the stomatal complex cells of the nectary epidermis was approximately 2 μm. The cuticular striae present close to the stomata were densely arranged. At the distance of 2 μm from the stoma, they were arranged collaterally towards the longer cell axis. At the opposite pole, the striae diverged slightly towards the neighbouring cells, forming rows with a radial pattern (Fig. 3b, f). The cuticular striae on the surface of each cell clustered into a single band with a width from 23 μm (‘Zabeliana’) to 32 μm (‘Schipkaensis’), and the differences were statistically significant (Table 2). Next to the stomatal complex, there were arcuately or radially arranged epidermis cells, which formed rows with other adjacent cells (Fig. 3a, b, d). In both cultivars, the number of bands formed by the cuticular striae in the stomatal complex corresponded to the number of cells (4–6) surrounding the stoma (Fig. 3b, f).

**Table 2:** Cuticle ornamentation on the surface area of the stomatal complex cells and the other epidermal nectary cells of two studied *P. laurocerasus* cultivars

Tested feature			Cultivar			
			‘Schipkaensis’		‘Zabeliana’	
			Min.–max.	Mean ± SD	Min.–max.	Mean ± SD
Stomatal complex cells						
Thickness of cuticular striae		(μm)	1.57–2.45	1.93 ± 0.29a	1.24–2.42	1.68 ± 0.33a
Distance between cuticular striae			1.11–3.38	2.12 ± 0.58a	1.31–3.44	2.09 ± 0.58a
Number of cuticular striae on the cell surface		(pc.)	4.00–8.00	6.69 ± 1.08a	4.00–10.00	7.25 ± 1.65a
Width of the band of cuticular striae		(μm)	26.23–36.58	31.56 ± 2.67a	18.50–28.85	23.28 ± 3.27b
The other epidermal cells						
Thickness of cuticular striae		(μm)	1.59–2.74	1.95 ± 0.28a	1.26–2.61	2.00 ± 0.42a
Distance between cuticular striae			0.98–2.85	1.86 ± 0.52a	1.41–2.92	2.03 ± 0.40a
Number of longitudinal cuticular striae on the cell surface		(pc.)	5.00–10.00	7.19 ± 1.60a	5.00–8.00	6.69 ± 0.95a
Width of the band of cuticular striae		(μm)	14.77–18.78	17.01 ± 2.21a	14.97–18.97	18.01 ± 2.80a
The diameter of the other epidermal cells	Min.	(μm)	22.88–53.50	37.45 ± 9.08a	25.58–37.41	32.73 ± 3.63a
	Max.		12.5–23.69	17.71 ± 2.89a	18.10–28.15	23.16 ± 3.14b

For the explanations, see Table 1

#### Other epidermal cells

**Fig. 4:**
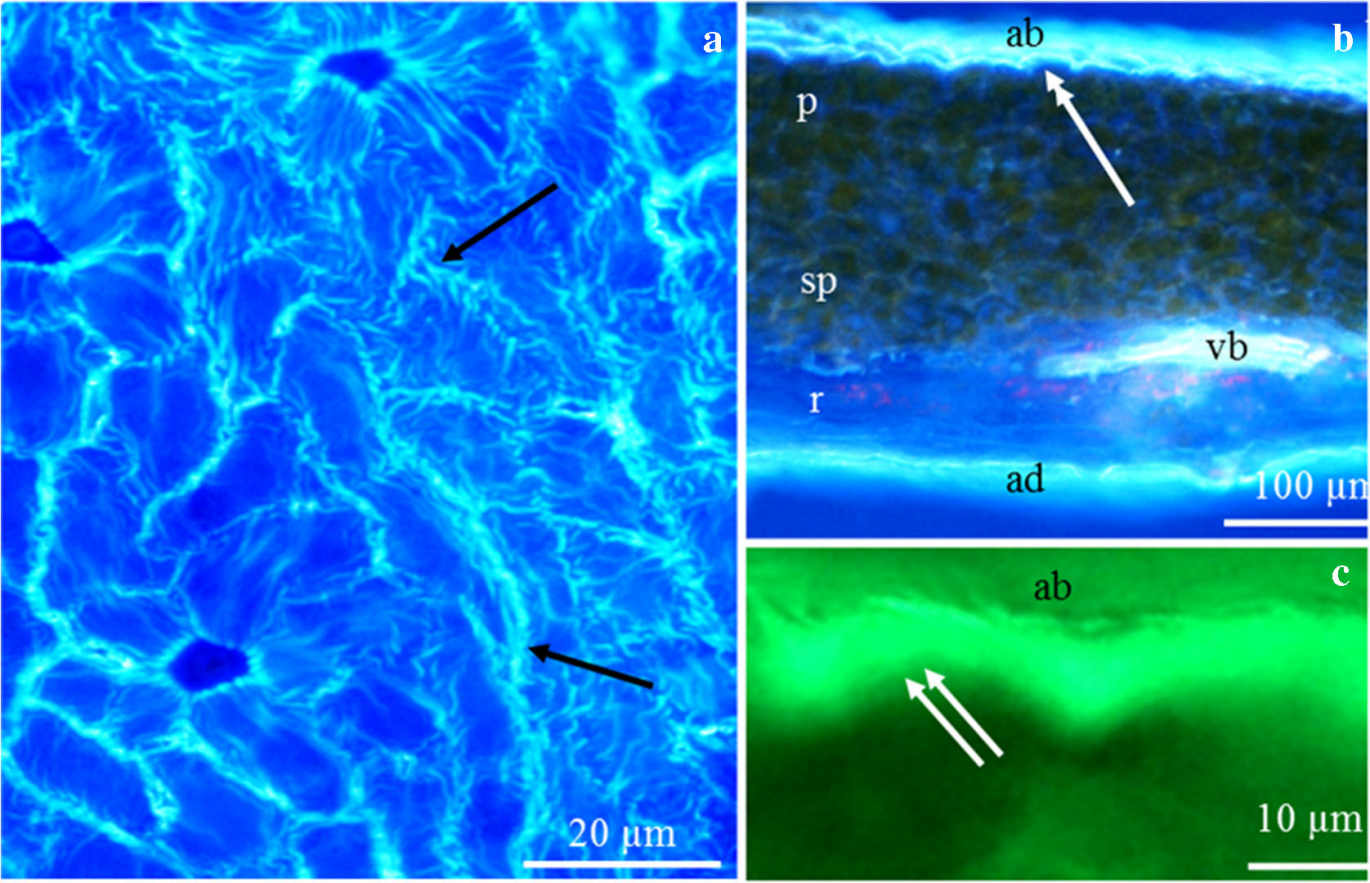
**a–c** Surface of the epidermis (**a**) and cross sections of the nectary (**b**, **c**) in *P. laurocerasus* ‘Schipkaensis’ (**a**, **c**) and ‘Zabeliana’ (**b**). **a** Stomatal complex and other epidermis cells, visible autofluorescence of cuticular striae (arrow). **b** Intense autofluorescence (double-headed arrow) of the nectary epidermis (ab), visible nectary parenchyma (p) and sub-nectary parenchyma (sp) cells, vascular bundle (vb) located on the border between the sub-nectary parenchyma and receptacle (r). **c** Epicuticular fluorescence (two arrows) of the nectary epidermis (ab). FM

The average thickness of the cuticular striae on the surface of these cells was 2 μm. The striae formed unidirectional bands with a width of 14.8–19 μm, with an average value of 17.5 μm. The cuticular ornamentation covered the entire surface of the epidermis cells, sometimes with one major stria (Fig. 3c). This pattern sometimes included several rows of cells with a visible band over the periclinal wall and with intertwined and discontinued striae (Fig. 3e). These structures, spaced 2 μm apart, were located along the longer axis of the cells. They were straight, arched, corrugated or zigzagged. On the surface of a single cell in both cultivars, there were approximately 7 striae (Fig. 3c, e; Table 2). These structures and the outer wall of epidermal cells of the nectary showed autofluorescence (Fig. 4a, b) and green epicuticular fluorescence in the presence of auramine O (Fig. 4c).

### Anatomy of the nectary

#### Nectary epidermis

**Fig. 5:**
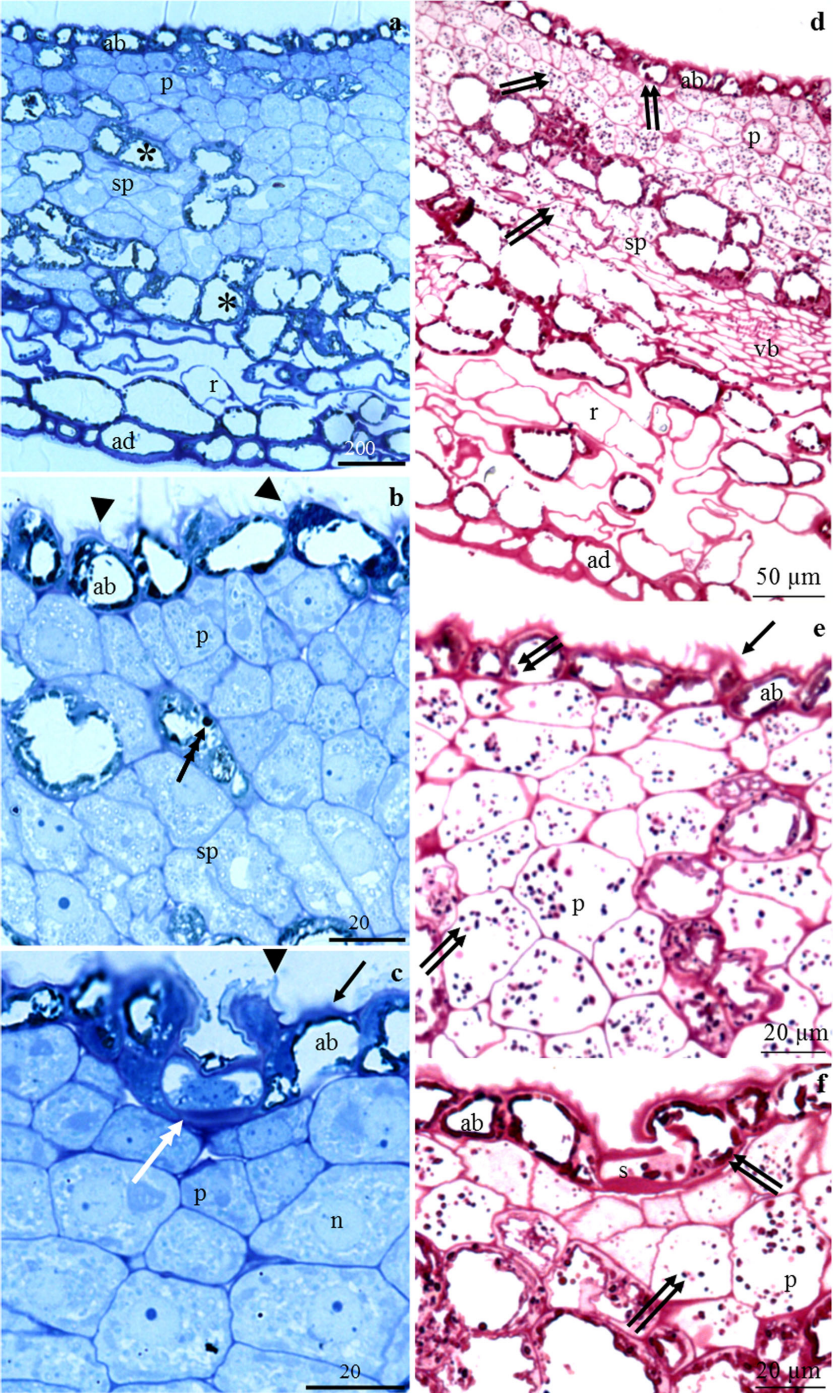
**a–f** Longitudinal sections of receptacle (**a**, **d**) and nectary cells (**a**–**f**) in *P. laurocerasus* ‘Schipkaensis’(**a**, **d**, **f**) and ‘Zabeliana’ (**b**, **c**, **e**). **a** Epidermal nectary cells (ab), tightly arranged nectary parenchyma cells (p), a layer of larger cells of sub-nectary parenchyma (sp), loosely arranged receptacle cells (pr). **b** Epidermal nectary cells (ab) with convexities of the outer cell wall (arrowhead), with more intensely coloured cytoplasm and various degrees of vacuolisation, nectary parenchyma cells (p) with dense cytoplasm, large nucleus and one or two nucleoli, large sub-nectary parenchyma cells (sp) with one or two small vacuoles, visible precipitates in the cell sap (triple-headed arrow). **c** Thick outer cell wall of epidermis (arrow), stomata (double-headed arrow) located below other epidermis cells, numerous vesicular structures in the nectary parenchyma cells (p), large centrally located nucleus (n) with nucleoli. **d** Pink stained cell wall polysaccharides, amyloplasts (two arrows) in the epidermal (ab) and parenchyma (p) nectary cells, visible vascular bundles (vb). **e** Intensely stained nectary epidermis cell walls, convexities (arrow) in the outer epidermis walls, amyloplasts (two arrows) in the parietal epidermis cytoplasm and numerous amyloplasts in the nectary parenchyma cells (p). **f** Amyloplasts (two arrows) in guard cells (s), in other epidermis cells (ab) and nectary parenchyma (p), visible protuberances of the outer cell wall (arrow). LM

The outer periclinal wall of the epidermis cells was thicker than the other walls and had different-sized convexities (Fig. 5a, b, e, f). The stomata were located below the other nectary epidermis cells (Fig. 5c, f). The protoplast of the nectary epidermis was darker and exhibited greater vacuolisation than the nectary parenchyma cells. There were dark amyloplasts in the cytoplasm (Fig. 5b). In the cross sections, the epidermis cells in the analysed cultivars were slightly elongated (14–17 μm). Their average height was 12 μm (Table 3).

**Table 3:** Characteristics of nectary and receptacle chosen anatomical features of two studied *P. laurocerasus* cultivars

Tested feature			Cultivar			
			‘Schipkaensis’		‘Zabeliana’	
			Min.–max.	Mean ± SD	Min.–max.	Mean ± SD
Nectary thickness		(μm)	175.82–236.74	200.38 ± 16.84a	119.31–180.12	152.57 ± 17.38b
Nectary epidermis cells						
Height	of nectary epidermis cells	(μm)	9.96–15.14	11.77 ± 1.72a	9.19–15.40	12.02 ± 1.89a
Length			12.12–21.40	16.48 ± 3.32a	10.12–18.95	13.60 ± 2.73a
Nectary parenchyma cells						
Number of layers	of the nectary parenchyma cells	(pc.)	3.00–5.00	3.69 ± 0.79a	2.00–4.00	2.50 ± 0.63b
Thickness of the layer		(μm)	63.49–106.12	79.31 ± 12.58a	30.01–53.29	38.84 ± 7.07b
Diameter			15.87–8.43	21.980 ± 3.46a	12.72–21.01	16.24 ± 2.44b
Subnectary parenchyma cells						
Number of layers		(pc.)	3.00–5.00	3.88 ± 0.50a	3.00–6.00	4.81 ± 1.05a
Thickness of the layer	of the subnectary parenchyma cells	(μm)	92.44–127.66	109.30 ± 11.35a	61.29–125.70	101.70 ± 18.64a
Diameter			23.11–39.11	28.62 ± 4.55a	15.48–29.69	21.45 ± 3.09b
Receptacle						
Height	of abaxial epidermis cells	(μm)	17.33–28.61	23.03 ± 3.15a	13.64–28.61	21.90 ± 4.12a
Length			30.84–52.29	41.24 ± 6.46a	17.46–36.27	25.11 ± 5.08b
Thickness of receptacle parenchyma cells			165.92–208.78	190.44 ± 12.67a	126.27–152.60	139.76 ± 7.30b
Thickness of the receptacle			188.86–233.99	213.47 ± 13.96a	145.01–170.91	161.67 ± 8.76b
Thickness of the receptacle with the nectary			377.60–438.20	413.85 ± 18.84a	275.07–351.03	314.24 ± 20.60b

For the explanations, see Table 1

#### Nectary parenchyma

The thickness of the layer of the nectary parenchyma cells was significantly higher in ‘Schipkaensis’ (79 μm) than in ‘Zabeliana’ (39 μm). The nectary parenchyma cells formed 2–5 rows with a diameter ranging from 16 to 22 μm in ‘Zabeliana’ and ‘Schipkaensis’, respectively. These differences were significant. The cells had a dense cytoplasm and a centrally located nucleus with a prominent darker nucleolus or two nucleoli. Numerous vesicular structures were visible in the cytoplasm. The protoplast contained a few small vacuoles or one larger vacuole (Fig. 5b, c). In the nectary parenchyma, there were single or a few cells located in a row and containing precipitates of calcium oxalate (Fig. 5b). Sometimes, these cells were present in the sub-nectariferous parenchyma.

The sub-nectary parenchyma consisted of 3–6 rows of cells. The thickness of this layer was in the range from 102 (‘Zabelina’) to 109 (‘Schipkaensis’) μm. The diameter of sub-nectary parenchyma cells was greater by approximately 25% compared to the nectar parenchyma cells (Table 3). The sub-nectary parenchyma cells had thinner walls and exhibited a greater diameter and a higher degree of vacuolisation than the nectary parenchyma cells (Fig. 5a).

In the receptacle, there were visible vascular bundles reaching the sub-nectary parenchyma cells and supplying the nectary (Fig. 5d). The receptacle thickness in ‘Zabeliana’ and ‘Schipkaensis’ differed significantly, i.e. 162 μm and 214 μm, respectively. In turn, the thickness of the *P. laurocerasus* nectary was in the range from 153 μm (‘Zabeliana’) to 200 μm (‘Schipkaensis’) and these differences were statistically significant (Table 3).

#### Polysaccharides in nectary cells

Insoluble polysaccharides, e.g. cellulose, hemicelluloses and pectins in the nectary epidermis and parenchyma cells gave a positive PAS reaction. In turn, plastids with starch granules were stained pink after addition of Schiff’s reagent. There were amyloplasts in the parietal cytoplasm of the nectary epidermis cells. These plastids were present in the cytoplasm of the stomatal guard cells. In the nectary parenchyma, they were located in the entire cross section of the cells (Fig. 5d–f). No amyloplasts were detected in the abaxial epidermis and receptacle parenchyma cells (Fig. 5d).

### Ultrastructure of the nectary

#### Nectary epidermis cells

**Fig. 6:**
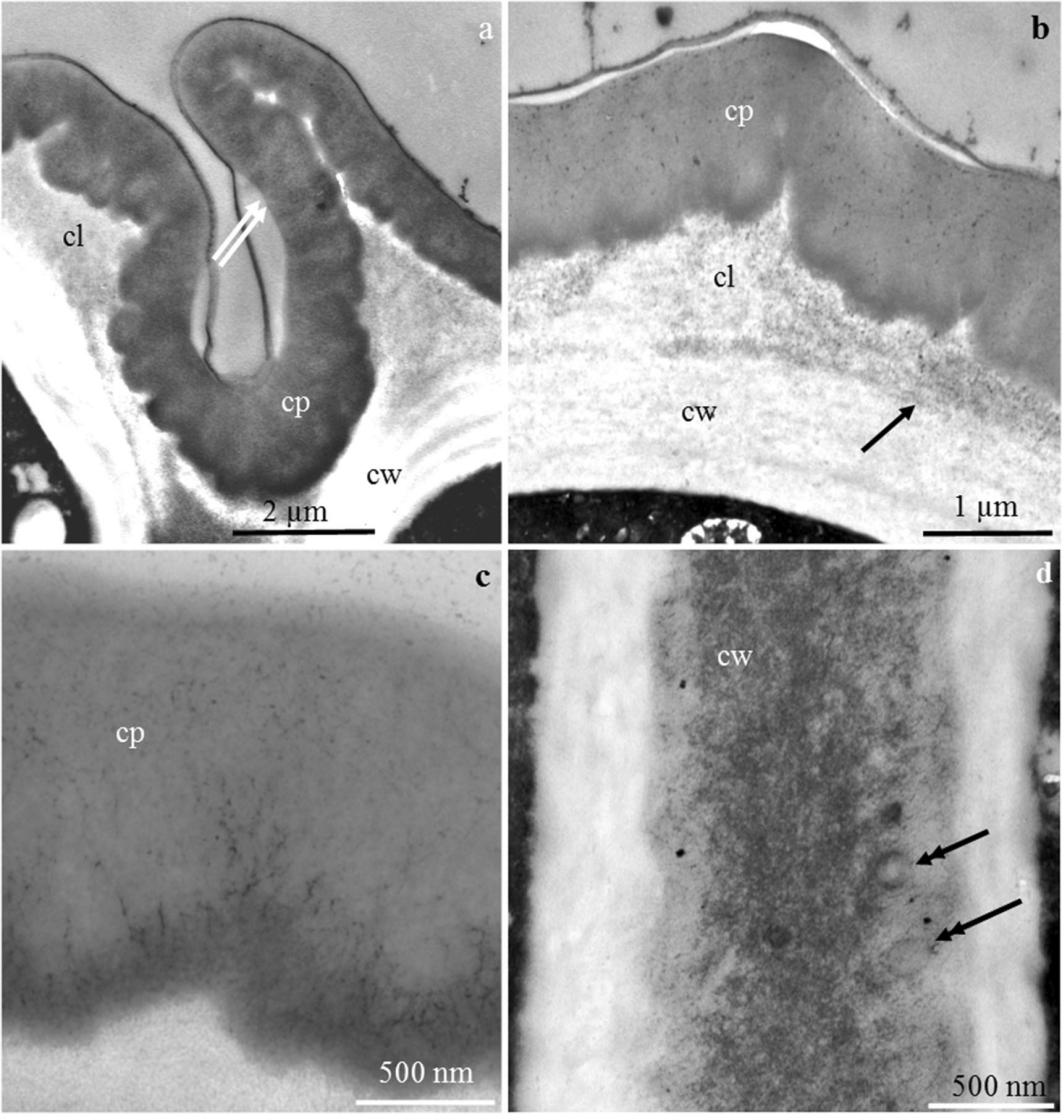
**a–d** The outer cell wall of nectary epidermis in *P. laurocerasus* ‘Schipkaensis’ (**a**, **c**) and ‘Zabeliana’ (**b**, **d**). **a** Outer periclinal cell wall, visible convexities of the wall (two arrows), lamellar (cp) and reticulate (cl) cuticle and the remaining part of the wall (cw). **b** Cuticle band uniformly distributed on the other part of the wall, visible lamellar (cp) and reticulate (cl) cuticle and pectin band (arrow). **c** Lamellar band of cuticle (cp). **d** Inner periclinal cell wall, visible vesicles in the wall (double-headed arrow). TEM

The outer periclinal cell wall of the epidermis in the floral nectaries of *P. laurocerasus* ‘Schipkaensis’ and ‘Zabeliana’ formed different-sized convexities with a varied outline on the longitudinal section (Fig. 6a). The cuticle layer in the examined cultivars formed a uniformly distributed, continuous, thick band (Fig. 6b) with a thickness within the range of 1–1.7 μm and a mean value of about 1.3 μm. The lamellar structure and the reticulate layer containing cellulose microfibrils were visible in the cuticle (Fig. 6a–c). The inner periclinal (0.7–1.2 μm) and anticlinal (0.45 μm) walls were substantially thinner than the outer periclinal epidermis walls (2.9–3.5 μm) (Table 4). In the anticlinal and periclinal cell walls, there were transport vesicles, probably involved in the apoplastic transport of prenectar substrates (Fig. 6d).

**Table 4:** Characteristics of the cell wall of nectary epidermis of two studied *P. laurocerasus* cultivars (μm)

Tested feature	Cultivar			
	‘Schipkaensis’		‘Zabeliana’	
	Min.–max.	Mean ± SD	Min.–max.	Mean ± SD
The thickness of the cuticle layer	1.13–1.67	1.30 ± 0.17a	1.02–1.46	1.21 ± 0.14a
Thickness of the remaining part of the outer periclinal cell wall (without cuticle)	1.54–2.55	1.90 ± 0.39a	1.5–2.19	1.7 ± 0.23a
Thickness of the outer periclinal cell wall	3.04–4.63	3.46 ± 0.56a	2.5–3.4	2.91 ± 0.26a
Thickness of the anticlinal cell wall	0.33–0.46	0.39 ± 0.04a	0.37–0.57	0.46 ± 0.06a
Thickness of the inner periclinal cell wall	0.86–1.55	1.16 ± 0.18a	0.56–0.81	0.71 ± 0.08b

For the explanations, see Table 1

**Fig. 7:**
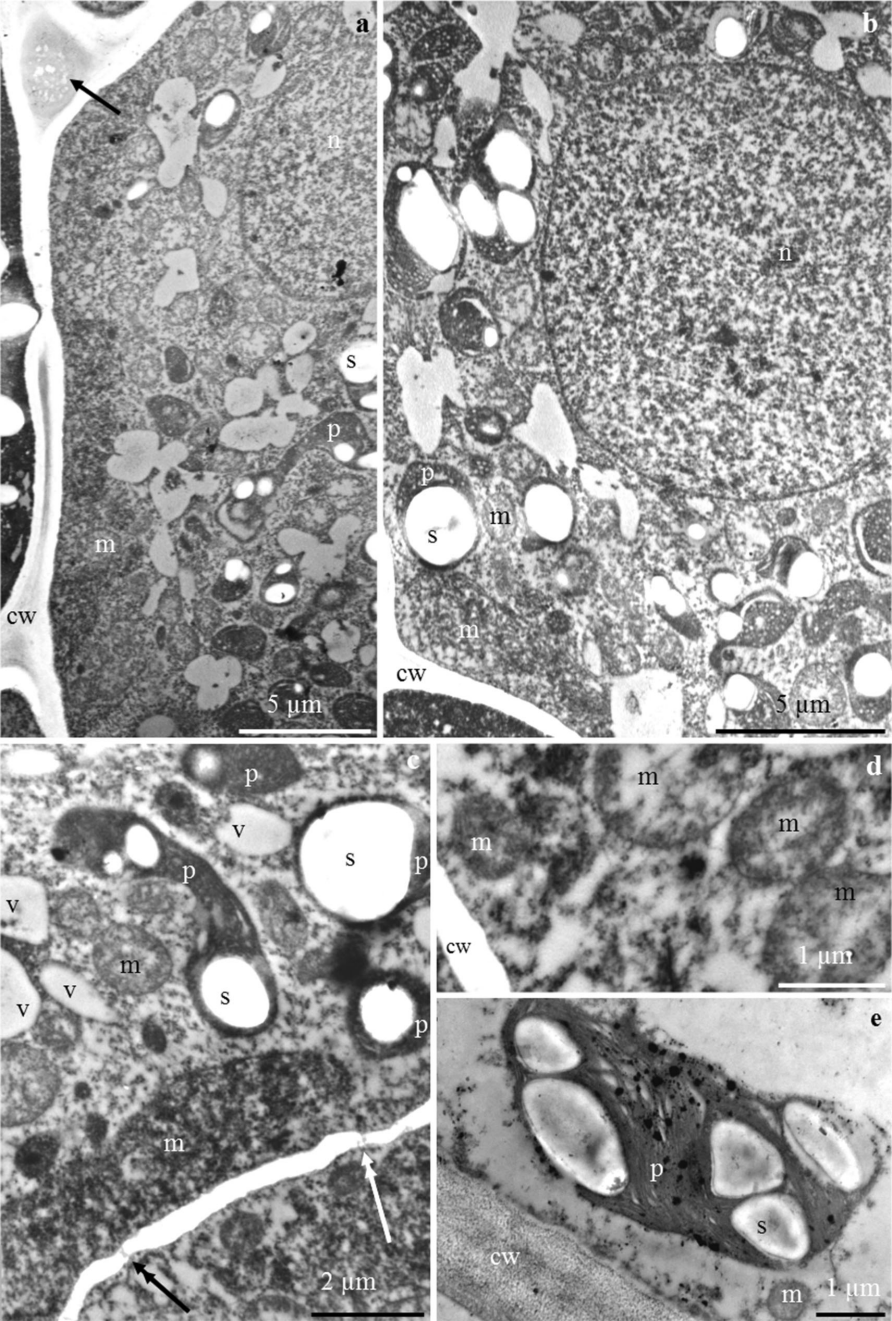
**a**–**f**. Nectary epidermis cells in *P. laurocerasus* ‘Schipkaensis’. **a**, **b** Electron dense protoplast, visible numerous plastids (p) with starch grains (s), mitochondria (m), spherical nucleus (n), and vesicular structures (arrow) in extracellular spaces (**a**). **c** Dense cytoplasm, visible pleomorphic plastids (p) with starch grains (s), mitochondria (m) located near the wall and plastids, small vacuoles (v), plasmodesmata in the periclinal cell wall (arrow with double arrowhead). **d** Mitochondria clustered near the cell wall (cw). **e** Plastid (p) with starch grains (s) located at the cell wall (cw), visible mitochondrion (m). TEM

In turn, an amorphous substance and lighter vesicular structures (Figs. 7a and 9d) were observed in the intercellular spaces. Plasmodesmata in contact with other cells were visible in the walls (Figs. 7c and 9c).

The protoplast of the nectary epidermis had electron-dense cytoplasm and a large centrally located spherical nucleus with a distinct nucleolus (Figs. 7a, b and 9c). Many pleomorphic mitochondria were located near the nucleus and in a parietal position. The mitochondria were arranged side by side or in groups and were sometimes arranged in a row close to the nucleus and the cell wall or surrounded by numerous plastids (Figs. 7b–d and 9c, d). Most plastids contained single, two, three, or sometimes even up to eight starch grains (Figs. 7b, c, e and 9a, b).

#### Nectary parenchyma cells

**Fig. 8:**
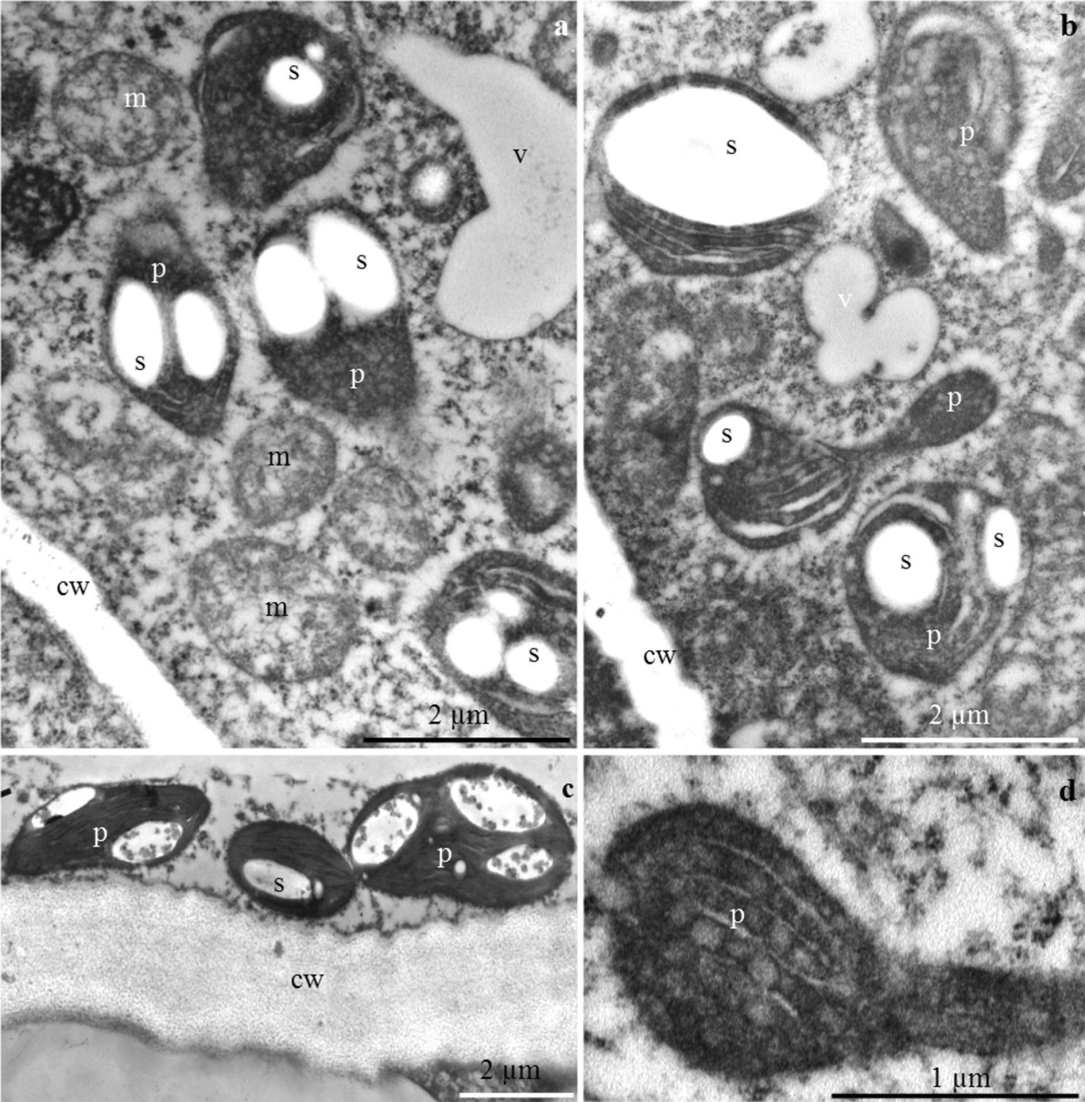
**a**–**d** Nectary parenchyma cells in *P. laurocerasus* ‘Schipkaensis’. **a**, **b** Numerous plastids (p) with starch grains (s), mitochondria (m) located close to amyloplasts and cell walls (cw), small vacuole (v). **c** Mitochondria (m), pleomorphic plastids (p) with starch grains (s), cell wall (cw). **d** Elongated plastid (p). TEM

The cell walls were sometimes corrugated and had a distinct middle lamella, likewise the epidermis cells (Fig. 10a–c). The cell nucleus was spherical or elongated and had electron-dense nucleoplasm (Fig. 10a, b, d). The cytoplasm contained numerous mitochondria with a well-formed internal structure (Figs. 8a and 10b, d). The mitochondria were arranged serially around the nucleus or were clustered near the plastids or cell walls (Figs. 8a and 10a–d). Large plastids with single or several starch granules were often detected in the cytoplasm of the nectary parenchyma. Various shapes of the plastids, besides typical ones, were noted: oblong and narrowed in the central part as well as elongated at one of the poles with a space visible in the thylakoid membrane system (Fig. 8a, b). The endoplasmic reticulum, i.e. the site of post-translational processes such as preparation for secretion, was tubular with circular (Fig. 10a) or arcuate (Fig. 10d) arrangement of tubules resembling characteristic compartments in the form of subcellular spherical structures (Fig. 10a). A low degree of protoplast vacuolisation was noted (Fig. 8a).

**Fig. 9:**
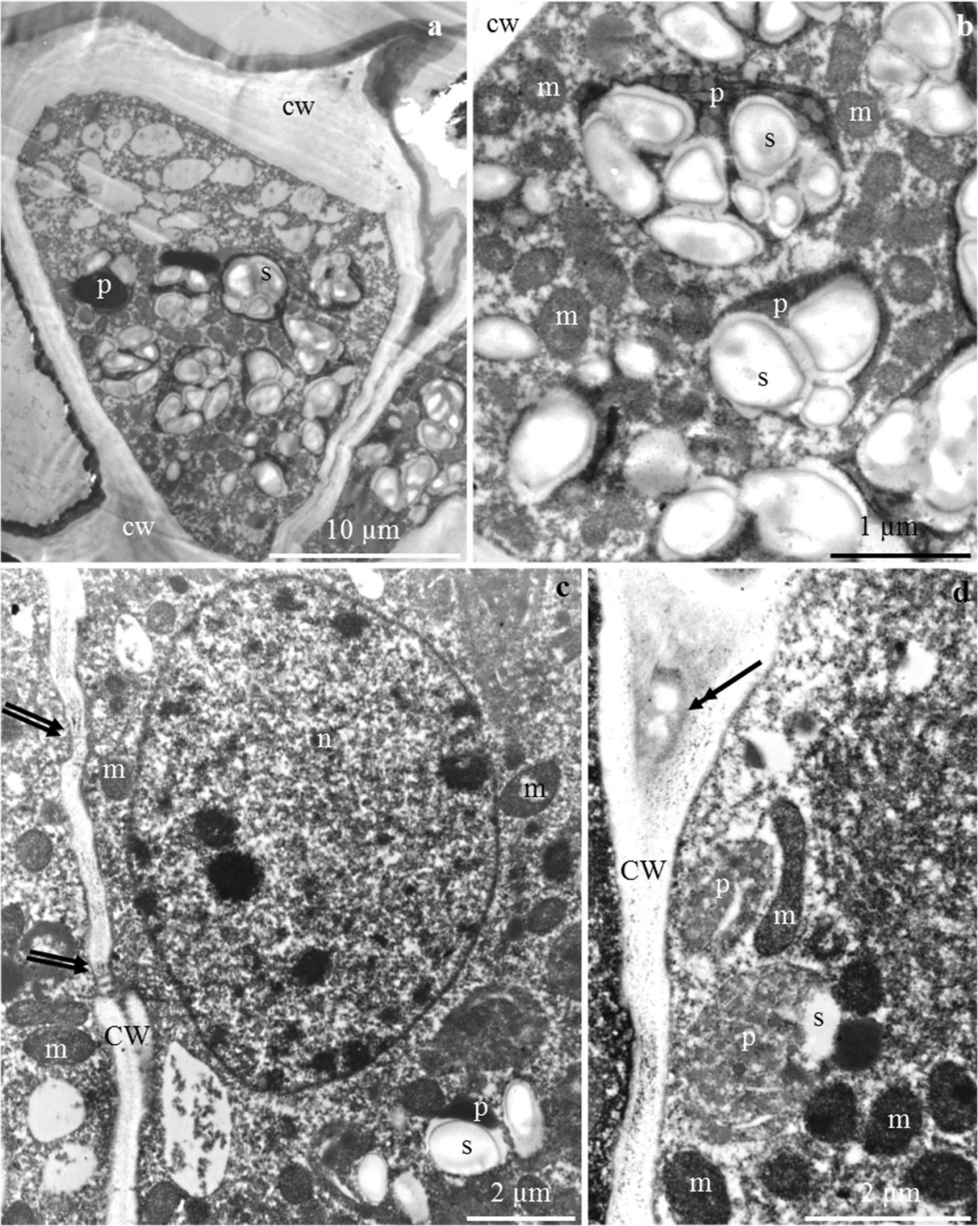
**a**–**d** Nectary epidermis cells in *P. laurocerasus* ‘Zabeliana’. **a** Thick outer cell wall (cw), visible protoplast with numerous starch grains (s) and small vacuoles (v). **b** Dense cytoplasm, numerous mitochondria (m) and plastids (p) with starch grains (s). **c** Plasmodesmata (two arrows) in the anticlinal cell wall (cw), large cell nucleus (n) with dense nucleoplasm, mitochondria (m) located close to the cell wall and nucleus, plastids (p) with starch grains (s). **d** Anticlinal cell wall (cw), visible vesicles (v) in the intercellular spaces (arrow with double arrowhead), clustered mitochondria (m). TEM

**Fig. 10:**
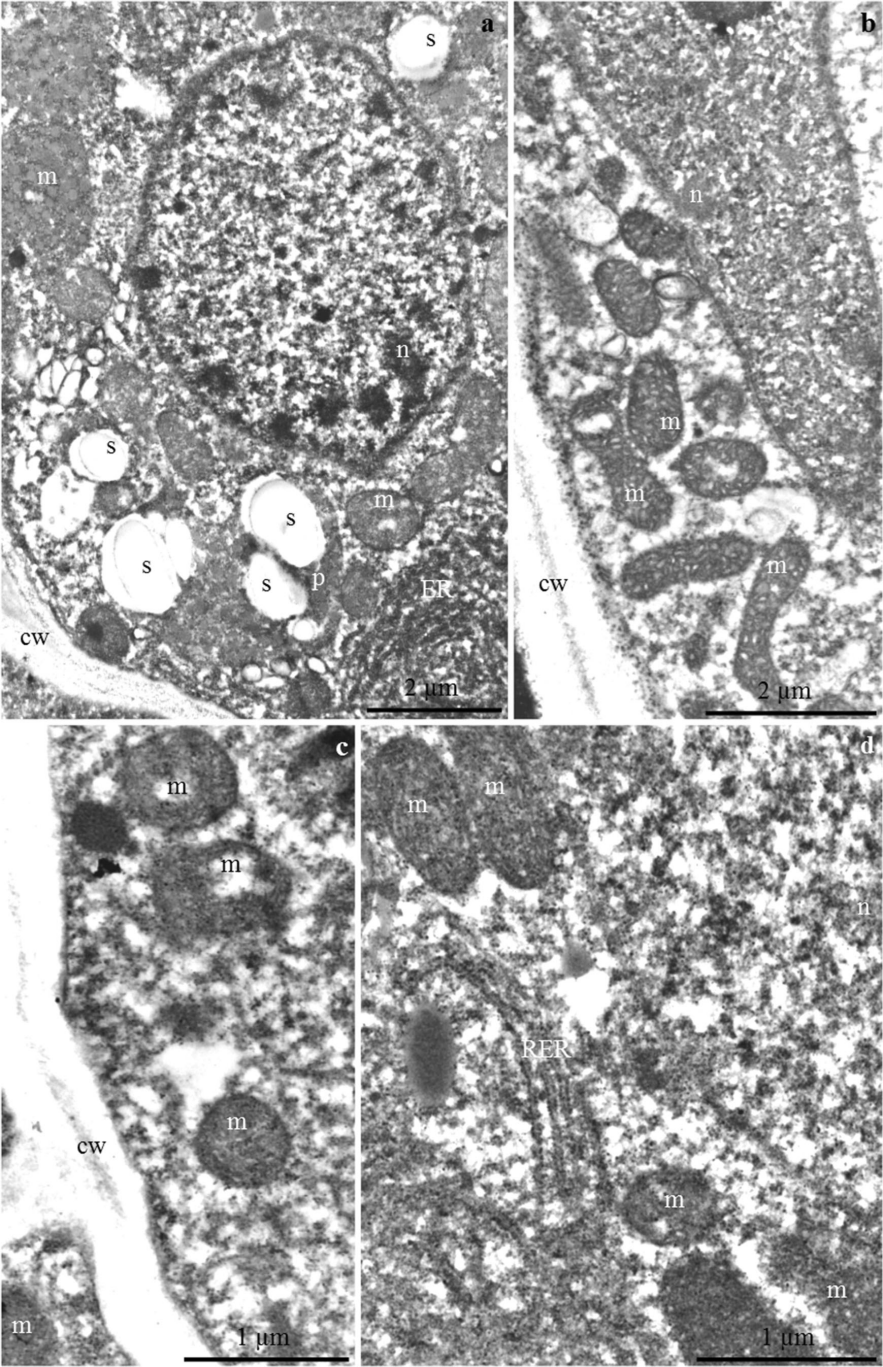
**a**–**d** Nectary parenchyma cells in *P. laurocerasus* ‘Zabeliana’. **a** Electron dense cytoplasm, plastids (p) with starch grains (s), numerous mitochondria (m), spherical cell nucleus (n), radially arranged ER. **b** Elongated cell nucleus (n), visible nucleolus, pleomorphic mitochondria (m) located at the cell wall and nucleus. **c** Serially arranged mitochondria (m) close to the anticlinal cell wall (cw). **d** Cell nucleus (n), coupled mitochondria (m), visible rough reticulum (RER). TEM

**Fig. 11:**
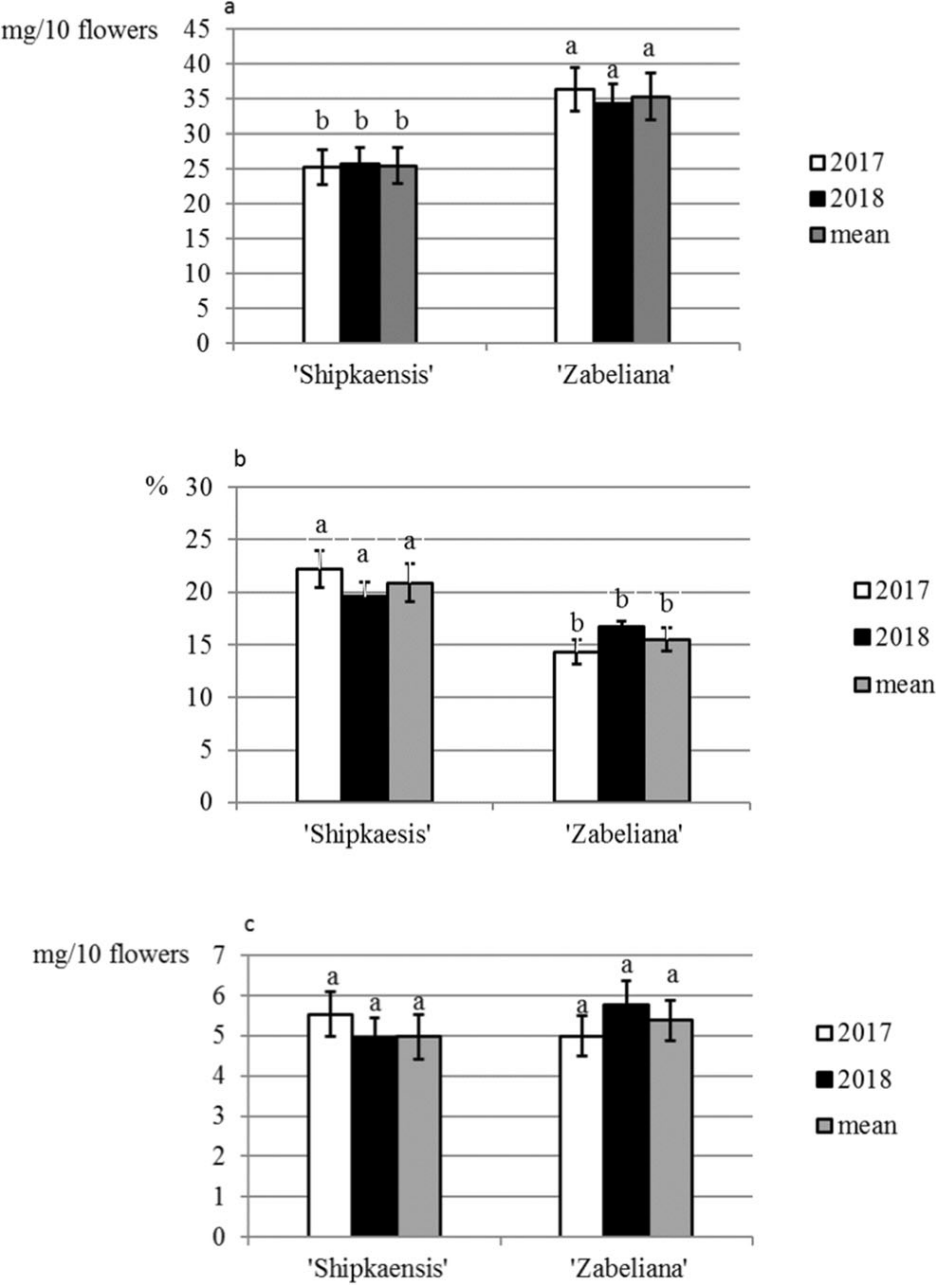
**a**–**c** Mass of nectar (a), concentration of sugars in nectar (b) and mass of sugar in nectar (c) in the studied cultivars of *P. laurocerasus*. Explanations: Mean values for each feature of nectar secretion abundance, calculated from twelve replications (*n* = 12), marked with the same letter are not different at *P* < 0.05 based on the HSD Tukey test. Vertical bars represent the standard deviation (SD) of means

**Fig. 12:**
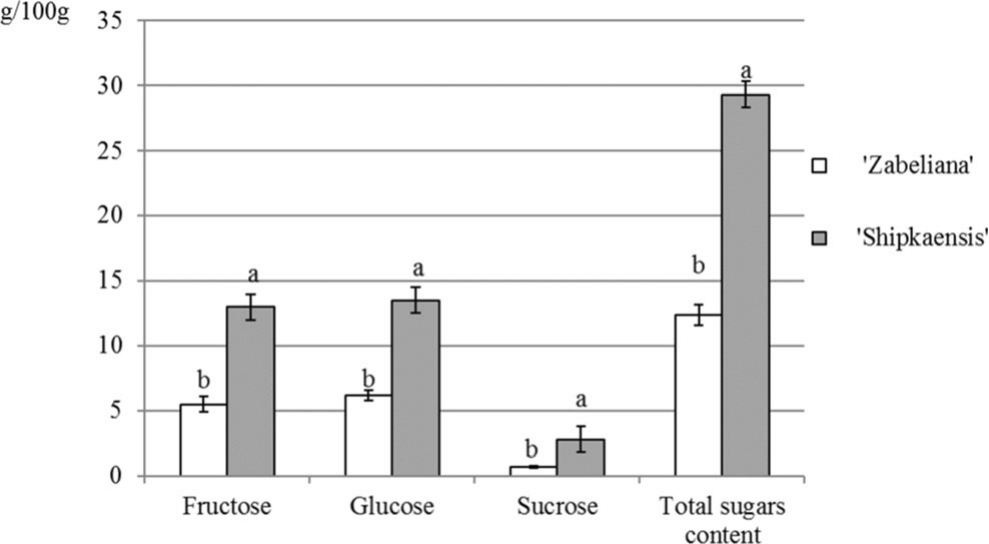
Total sugar content and percentage of sugars: glucose, fructose and sucrose in the nectar of the two cultivars *P. laurocerasus* studied*.* Explanations: Mean values for quantitative and qualitative composition of sugars in the nectar, calculated from three replications (*n* = 3), marked with the same letter are not different at *P* ≤ 0.05 based on the HSD Tukey test. Vertical bars represent the standard deviation (SD) of means

### Abundance of nectar secretion

Nectar secretion was noted already in the bursting bud phase, as there were small secretion droplets on the nectary surface. Single *P. laurocerasus* flowers secreted 2.5–3.5 mg of nectar. The concentration of sugars in the nectar was in the range of 16% (‘Zabeliana’)–26% (‘Schipkaensis’). The weight of sugars in the nectar was 5–5.4 mg per flower (Fig. 11a–c). It was found that the secretion of nectar and sugar mass in the nectar was much higher in the ‘Zabeliana’ flowers with a thinner layer of secretory active cells compared to the production of the secretion by the thicker nectary of ‘Schipkaensis’.

The qualitative analysis of the sugar composition revealed that the nectar of the two cherry laurel cultivars was dominated by glucose (G), followed by fructose (F) and the lowest content of sucrose (S). The S/(G + F) ratio was 0.06 (Fig. 12). This value indicates that the nectar can be classified into a group with a dominant concentration of hexoses. The abundance of nectar production and the content of the analysed sugars in this secretion were higher in ‘Schipkaensis’ than in ‘Zabeliana’. *Prunus laurocerasus* flowers were visited mainly by honeybees and bumblebees.

## Discussion

### Micromorphology of the nectaries

#### Colour of the nectaries

The nectaries analysed in the *P. laurocerasus* cultivars represent the receptacular type. This type of nectary, specified in accordance with the classification proposed by Bernardello (2007), has been described in other species of *Prunus* (Radice and Galati 2003; Chwil 2013). The cells changed their colour from intense orange in the initial secretion phase to light yellow in the full secretion phase. A similar metabolic variability of the nectary colour during consecutive secretion phases was observed in different species of this genus (Chwil 2013). This phenomenon is a result of differentiation of chloroplasts and biosynthesis of pigments from the group of carotenoids (β-carotene, adonixanthin, adonirubin, astaxanthin, canthaxanthin and violaxanthin) and anthocyanins (Mann et al. 2000; Horner et al. 2007; Paiva 2012). The change in the nectary gland colour proceeded from the inner portion towards the epidermis. During the biosynthesis of pigments in nectary cells, chloroplasts with well-developed thylakoid membranes accumulate starch and, as the concentration of carotenoids increases, they undergo transformation into amylochromoplasts and serve the function of chromoplasts in the mature gland stage (Horner et al. 2007; Pacini et al. 2003; Liu and Thornburg 2012).

#### Cuticular ornamentation

Similar striation of the nectary epidermis cuticle to that observed in the cherry laurel cultivars examined in the present study was described by other authors in *P. armeniaca*, *P. avium*, *P. cerasus*, *P. domestica* and *P. persica* (Farkas and Zajácz 2007; Liu and Zhao 2011; Chwil 2013). In turn, the nectaries in *P. communis* were characterised by a smooth cuticle surface (Farkas and Zajácz 2007). Orosz-Kovács et al. (1990) reported transition between the reticulate and grooved types of the *P. laurocerasus* cuticle. The varied cuticle ornamentation is one of the taxonomic criteria differentiating species in many families (Orosz-Kovács et al. 1990; Chwil et al. 2006; Ganeva and Uzunova 2010; Tahir and Rajput 2009; Chwil and Weryszko-Chmielewska 2011). Cuticular striae are involved in the transport of secreted nectar to the accumulation site and protect the secretion from evaporation (Orosz-Kovács and Apostol 1996). The glossy stripes reflecting the sunrays are a light signal determining the entomological attractiveness of flowers (Juniper and Jeffree 1983; Harborne 2014; Wu et al. 2011). The multifunctional cuticle layer prevents water loss, protects from abiotic environmental factors (Serrano et al. 2014; Wu et al. 2011), microbial infestation and pest attack (Schreiber et al. 2005; Domínguez et al. 2017). At higher temperatures, the molecular structure of the wax layer changes, which consequently leads to increased transpiration intensity (Schreiber 2001, 2002, 2010).

#### Stomata

Closed, semi-open and open stomata were observed in the nectary epidermis of the two studied cultivars of *P. laurocerasus* as well as in other species *Prunus* (Chwil 2013). These structures were distributed over the entire nectary surface. Davis and Gunning (1992) reported that stomata did not have the ability to regulate the size of the aperture during nectar secretion and the opening width of stomatal cuticular ledge was not associated with the secretory stage. As suggested by Orosz-Kovács (1993), the variation of the aperture width is a consequence of rhythmic nectar secretion.

The number of stomata per 1 mm^2^ of the nectary epidermis in the analysed cherry laurel (45–71) was similar to that observed in *P. cerasus* and two or three times higher than in *P. armeniaca*, *P. avium*, *P. domestica* and *P. persica* (Chwil 2013). As suggested by Pearce et al. (2005), the different densities of stomata per unit area may be related to adaptation of the taxon to environmental conditions.

The actinocytic type of stomata, similar to that present in the cherry laurel, has been described in the leaf epidermis of several taxa of *Prunus* (Marrero and Nogales 2005), in turn, other described taxa of *Prunus* exhibited varied location of stomata relative to the epidermis level. They were located at the same level, below or above the epidermis in *P. domestica* and at the same level and below the epidermis in *P. communis* (Farkas and Zajácz 2007). As shown by Orosz-Kovács et al. (1996), the location of stomatal cells at or below the level of the epidermis indicates the meso- and xeromorphic type of nectary, whereas stomata located above the epidermis level represent the hygromorphic type.

#### Stomatal complex

The stomata described in the present study were surrounded by 4–10 radially arranged adjacent cells. They formed a stomatal complex with a diameter of 61–66 μm. The size of the stomatal complex, the topography of stomata and trichomes are diagnostic traits. These parameters are used for differentiation of closely related taxa not only in the complex taxonomy of *Prunus* but also in other systematic categories, e.g. in the family Rosaceae (Song and Hong 2014).

### Anatomy of the nectary

The nectaries of the analysed *P. laurocerasus* cultivars were made up of a single-layered epidermis and 3–5 rows of nectary parenchyma cells with a thickness of the layer in the range of 39–79 μm. The values of these parameters were lower than in other *Prunus* species (Chwil 2013). In turn, the cells of this gland in *P. persica* formed several layers (Radice and Galati 2003).

The nectary epidermal cells examined in the present study had a thicker and convex outer wall and a higher degree of vacuolisation than the nectary parenchyma cells. The active secretory cells exhibited a large nucleus and darker protoplast. Cells that had just undergone division were present between the fully developed nectary parenchyma cells. The development of the nectary tissue was accompanied by increased vacuolisation and the cytoplasm contained vesicular bodies. In the literature, there are only scanty data on the anatomical structure of the nectary in *Prunus* (Radice and Galati 2003; Chwil 2013). Two types of nectary parenchyma cells have been distinguished. The first type accumulated mainly chlorophyll and small amounts of photosynthetic starch. The other type of parenchyma cells stored photosynthetic starch every day for several days, and these cells accumulated nectar as well as starch (Pacini et al. 2003).

The presence of numerous plastids was detected in the protoplasts of the nectary epidermis and parenchyma in the analysed *P. laurocerasus* cultivars and other taxa in the genus (Radice and Galati 2003; Chwil 2013). The dynamics of their formation is related to the development of the nectary tissue (Nepi et al. 1996a, b). In the present study on *P. laurocerasus* cultivars, the presence of starch grains in the plastids was shown. Similar occurrence of amyloplasts was reported in *P. persica* (Radice and Galati 2003). As shown by literature reports, the amyloplasts in the nectary parenchyma cells were larger and more numerous and contained a larger number of smaller starch grains than in the epidermis cells. Formation of small starch grains is more beneficial due to the larger area of exposure to enzymes (Nepi et al. 1996a, b). The formation of starch grains in amyloplasts is a result of metabolic transformations with the involvement of four groups of enzymes: ADP-glucose pyrophosphorylase, starch synthase, starch-branching enzymes and starch-debranching enzymes (Ren et al. 2007a, b). Differentiation of plastids in nectary epidermis cells occurs 4 days later than in parenchyma cells (Nepi et al. 1996a). The process of formation of amyloplasts was enhanced from the early development stages of the nectary to the stage of maximum starch accumulation and ceased before anthesis. Transient starch in the nectaries is the main source of nectar carbohydrates. Starch metabolism in the nectary cells provided substrates for the production of nectar sugars and regulated the inflow of sugar to nectaries in a specific development phase (Ren et al. 2007a).

### Ultrastructure of the nectary

The thickness of the outer cell walls of the *P. laurocerasus* epidermis (2.9–3.5 μm) shown in this study was approximately three times as high as in several species of *Prunus* (0.9–1.3 μm) (Chwil 2013). The thickness of the cuticle layer on the *P. laurocerasus* nectary epidermis surface (1.3 μm) was higher than the range of 0.4–0.8 μm reported for the leaves of this species (Kirsch et al. 1997).

Numerous plasmodesmata were observed in the present study in epidermal nectary cells as well as in the walls of adjacent parenchyma cells. The presence of a larger number of plasmodesmata indicates a symplastic flow of prenectar. The desmotubules of the plasmodesmata were linked with the ER of adjacent cells, while the cytoplasmic annulus between the desmotubule and the inner face of the plasma membrane was parallel sided and non-constricted (Gunning and Hughes 1976; Gunning and Robards 1976). The continuity of protoplasts determines the symplastic transport of prenectar during the development of the nectary (Sawidis et al. 1987; Paiva and Machado 2008; Paiva 2009). The vesicles present between the fibrillar systems of the anticlinal and periclinal walls in the nectary cells in the two *P. laurocerasus* cultivars are probably involved in the apoplastic transport of prenectar substrates. The nectary cells in *P. persica* can participate in symplastic and apoplastic nectar transport (Radice and Galati 2003).

The present study indicates the occurrence of a lighter vesicular structure and an amorphous substance in the intercellular spaces. Amorphous material has also been observed in the nectaries of different species of various genera. The substance was a residue of secretion accumulated in the periplasmic space, and its quantity increased during anthesis (Paiva and Machado 2008; Paiva 2009: Chwil and Chwil 2012).

The different ER profiles observed in the epidermis and parenchyma nectary cells of the studied cherry laurel cultivars were characterised by a parallel, semicircular or circular arrangement of tubules. The configuration and quantity of ER depend on the metabolic activity and the stage of cell development. The RER profiles were concentrated near the plasmalemma. A higher density of ER indicates active metabolic processes in cells (Fahn 1979, 1988, 2000; Nepi 2007; Paiva 2009). The density of ER increases in the subsequent phases of nectary development (Paiva and Machado 2008; Kram et al. 2009).

The presence of numerous mitochondria with a well-developed system of internal membranes in the active secretory cells detected in this study evidences the secretory activity of these cells (Wist and Davis 2006; Nepi 2007; Paiva and Machado 2008). The number of mitochondria increases simultaneously with the development of amyloplasts in the nectary epidermal and parenchyma cells (Nepi et al. 1996a). This is closely related to the increased demand for energy required of nectar production and starch hydrolysis (Southwick 1984; Pyke 1991; Paiva and Machado 2008). Numerous amyloplasts, mitochondria and an extensive endoplasmic reticulum in the active secretory cells indicates high transduction of energy required for degradation of starch and synthesis of secretory proteins (Liu and Zhao 2011).

The extensive system of mitochondrial inner membranes, the endoplasmic reticulum with varied arrangement of tubules and the numerous transporting vesicles observed near the plasmalemma in the present study suggest granulocrine nectar secretion. A similar mode of nectar secretion is characteristic for many species of various genera (Chwil and Chwil 2012; Ojeda et al. 2014; Kowalkowska et al. 2018). The floral nectaries exhibited expanded ER cisterns and large vesicles associated mainly with the *cis*-side of dictyosomes. The secretion was transported inside the vesicular structures and, next, through the fusion of vesicles with the plasmalemma, the excretion was accumulated in the subcuticular space. The irregular outline of the plasmalemma with numerous vesicles determined the granulocrine secretion of nectar into the periplasmic space and then onto the outer surface (Chwil and Chwil 2012; Kowalkowska et al. 2012, 2015, 2018).

### Nectar secretion

In the studied cultivars of *P. laurocerasus*, no positive relationships were found between the size and the number of stomata per surface unit and the mass of secreted nectar. The 16–21% concentration of sugars in the *P. laurocerasus* nectar was lower than the value of 25–35% estimated in *P. armeniaca*, *P. persica*, *P. cerasus*, *P. avium* and *P. domestica* (Orosz-Kovács et al. 1995; Horváth and Orosz-Kovács 2004; Farkas and Zajácz 2007; Chwil 2013). The *P. laurocerasus* nectar represented the class with a dominant concentration of hexoses. The nectar of *P. laurocerasus*, *P. spinosa*, *P. persica* and *P. domestica* was dominated by fructose and glucose, whereas sucrose dominated in *P. avium*, *P. concinna*, *P. incisa* and *P. speciosa* (Percival 1961). The nectar of *P. armeniaca* and *P. persica* represents a group with dominance of hexoses, the nectar of *P. domestica* was rich in hexoses and sucrose dominated in the nectar of *P. cerasus* and *P. avium* (Chwil 2013). The proportion and concentration of sugar components in nectar are the bases of bees’ nectar preferences (Bukovics et al. 2003). The variable quantity and quality of nectar in different species of *Prunus* were associated with the individual and structural traits of a taxon, as well as with cyclic nectar secretion (Gupta et al. 1990; Bordács et al. 1995; Orosz-Kovács et al. 2000; Horváth and Orosz-Kovács 2004). The quantity and quality of nectar were determined by the stage of flower development, nectar resorption and wide availability for insects. The production of sugar in the nectar was intensified after the visits by pollinators (Torres and Galetto 1998). The alterations in the amount and quality of nectar composition were determined by hour-related differences in the species composition of visiting insects, by the cyclic mode of secretion and meteorological conditions. Honeybees’ visits in the flowers were intensified in wet weather due to the large amount of readily available nectar (Corbet 1978). Higher humidity increased the nectar volume and doubled the sucrose level in the flowers (Wyatt et al. 1992). Investigations conducted by Petanidou et al. (2006) confirm that nectar secretion determines the preferences of pollinators. Phenylalanine and gamma-aminobutyric acid exerted a strong impact on the number of visits, especially in the case of bees and flies, and the nectar volume was positively correlated with the number of bees and negatively correlated with the number of flies.

## Conclusions

The micromorphological structure of the nectaries in ‘Schipkaensis’ exhibited denser (approx. 39%) and larger (approx. 50%) stomata and thicker (approx. 13%) cuticular striae forming wider bands (approx. 26%) than in ‘Zabeliana’. The cuticular ornamentation and the topography of stomata on the surface of the *P. laurocerasus* nectary epidermis are helpful in differentiation between closely related taxa of *Prunus*. The anatomical features of the nectary epidermis and parenchyma as well as the ultrastructural traits of the epidermis had higher values in the ‘Schipkaensis’ nectaries compared with ‘Zabeliana’. The results of this investigation provide new data on features of *P. laurocerasus* epidermal micromorphology, tissue anatomy and ultrastructure of floral nectary cells as well as nectar abundance and sugar composition. Moreover, the abundance of nectar production and the quantitative and qualitative compositions of nectar determine the nutritional value of nectar for pollinating insects and can be used in honey industry (beekeeping, honey production process, and honey consumers). Our investigations revealed a high apicultural value of *P. laurocerasus* and a higher nutritional value of the *‘*Schipkaensis’ nectar than ‘Zabeliana’. With its long and abundant flowering as well as the amount of produced nectar, the cherry laurel can be a valuable entomophilous species recommended for cultivation in nectariferous zones, which can be insect pollinator refuges in urban and rural areas; however, climatic conditions eliminating the invasiveness of these plants should be considered. Further assessment and research on biological diversity in the context of genetic species and ecosystem diversity are needed. The knowledge at the plant-pollinator interaction level in pollination ecology and biochemistry is also necessary.
